# The clinical effectiveness of intensive management in moderate established rheumatoid arthritis: The titrate trial

**DOI:** 10.1016/j.semarthrit.2020.07.014

**Published:** 2020-10

**Authors:** David Scott, Fowzia Ibrahim, Harry Hill, Brian Tom, Louise Prothero, Rhiannon R. Baggott, Ailsa Bosworth, James B. Galloway, Sofia Georgopoulou, Naomi Martin, Isabel Neatrour, Elena Nikiphorou, Jackie Sturt, Allan Wailoo, Frances M.K. Williams, Ruth Williams, Heidi Lempp

**Affiliations:** a Centre for Rheumatic Diseases, Department of Inflammation Biology, School of Immunology and Microbial Sciences, Faculty of Life Sciences and Medicine, King's College London Cutcombe Road, London, SE5 9RJ, United Kingdom; b ScHARR Health Economics and Decision Science, The University of Sheffield, Regent Court, 30 Regent Street, Sheffield, S1 4DA, United Kingdom; c MRC Biostatistics Unit, University of Cambridge, Cambridge Institute of Public Health, Forvie Site, Robinson Way, Cambridge Biomedical Campus, Cambridge, CB2 0SR, United Kingdom; d National Rheumatoid Arthritis Society (NRAS), Switchback Office Park, Gardner Rd, Maidenhead, SL6 7RJ, United Kingdom; e Department Of Adult Nursing, Florence Nightingale Faculty of Nursing, Midwifery & Palliative Care, King's College London, James Clerk Maxwell Building, 57 Waterloo Road, London SE1 8WA, United Kingdom; f Twin Research & Genetic Epidemiology, School of Life Course Sciences, King's College London, St Thomas' Hospital, London SE1 7EH, United Kingdom

**Keywords:** Intensive management, Rheumatoid arthritis, Clinical trial, Anti-rheumatic agents, Person-centred care, Psychosocial support

## Abstract

**Objectives:**

Many trials have shown that intensive management is effective in patients with early active rheumatoid arthritis (RA). But its benefits are unproven for the large number of RA patients seen in routine care who have established, moderately active RA and are already taking conventional synthetic disease modifying anti-rheumatic drugs (csDMARDs). The TITRATE trial studied whether these patients also benefit from intensive management and, in particular, achieve more remissions.

**Methods:**

A 12-month multicentre individually randomised trial compared standard care with monthly intensive management appointments which was delivered by specially trained healthcare professionals and incorporated monthly clinical assessments, medication titration and psychosocial support. The primary outcome was 12-month remission assessed using the Disease Activity Score for 28 joints using ESR (DAS28-ESR). Secondary outcomes included fatigue, disability, harms and healthcare costs. Intention-to-treat multivariable logistic- and linear regression analyses compared treatment arms with multiple imputation used for missing data.

**Results:**

459 patients were screened and 335 were randomised (168 intensive management; 167 standard care); 303 (90%) patients provided 12-month outcomes. Intensive management increased DAS28-ESR 12-month remissions compared to standard care (32% vs 18%, *p* = 0.004). Intensive management also significantly increased remissions using a range of alternative remission criteria and increased patients with DAS28-ESR low disease activity scores. (48% vs 32%, *p* = 0.005). In addition it substantially reduced fatigue (mean difference -18; 95% CI: -24, -11, *p*<0.001). There was no evidence that serious adverse events (intensive management =15 vs standard care =11) or other adverse events (114 vs 151) significantly increase with intensive management.

**Interpretation:**

The trial shows that intensive management incorporating psychosocial support delivered by specially trained healthcare professions is effective in moderately active established RA. More patients achieve remissions, there were greater improvements in fatigue, and there were no more harms.

## Introduction

Despite the increasing availability of biological and other innovative therapies, rheumatoid arthritis (RA) remains a major health problem [1], [2], [3]. Current treatment recommendations in clinical guidelines advocate intensive management using treat-to-target strategies [4, 5]. There is strong evidence these are effective in early RA patients with high disease activity [6], [7], [8], [9]. However, a large number of patients followed in specialist units who are receiving conventional synthetic disease modifying anti-rheumatic drugs (csDMARDs) continue to have moderately active established RA [10, 11]. Despite the known poor long-term outcomes of these patients, there is uncertainty whether they too will benefit from intensive management.

The TITRATE trial took place in routine care settings across multiple centres with management delivered by specialist nurses and other health care professionals who had completed a targeted two day training programme. It evaluated the effectiveness of intensive management in moderately active established RA. Based on monthly clinical assessments drug therapy was optimised and patients also received psycho-social support delivered by trained nurses and other practitioners using motivational interviewing techniques [12]. One previous trial in such patients, the British Rheumatoid Outcome Study Group (BROSG), undertaken in the pre-biological era [13]; found intensive management using csDMARDs achieved only modest increases in remission and the differences were not significant. North American experience with similar patients has been variable. Harrold et al. found no benefit from treat to target approaches [14]. In contrast studies by Solomon et al. found benefit after training clinicians involved in general principles of management [15], [16], [17]. Uncertainty about the benefits of intensive management in moderately active established RA remains an important challenge when generalising treat-to-target approaches [18].

The TITRATE trial bridges this evidence gap by testing the hypothesis that 12-months of intensive management in patients with established moderately-active RA given in conjunction with psychosocial support provides more remissions than standard care.

## Patients and methods

### Design

An open-label, 12-month, pragmatic, randomised, multicentre, two-arm, parallel group superiority trial.

### Participants

Patients were recruited from 39 English rheumatology centres. Included patients comprised: males and females over 18 years; who met 2010 RA classification criteria [19]; had received ≥6 months csDMARDs; were currently receiving at least one csDMARD; had moderate/ intermediate disease activity (DAS28-ESR 3.2–5.1 with three or more swollen and/or tender joints out of 66/68 and at least one swollen joint); were able and willing to follow intensive management. Patients were excluded who had comorbidities making treatment intensification inadvisable (e.g. heart failure); had failed ≥5 csDMARDs; received biologics; had irreversible disability from extensive joint damage; were pregnant, breast-feeding or women planning to conceive; had recently participated in another clinical trial; and were currently on early RA management pathway.

### Interventions

Drug treatment complied with guidance from the National Institute for Health and Clinical Excellent (NICE) and the national specialist society (British Society for Rheumatology).

*Standard Care:* clinicians followed local pathways for managing moderate disease activity patients reflecting national guidance [20, 21] without specific treatment and follow-up plans.

The approach for managing RA in England when the trial was undertaken involved optimising csDMARD monotherapy, considering combination therapy with csDMARDs, and giving biologics if patients had active disease despite failing to respond to two csDMARDs.

*Intensive Management:* this was delivered by nurses and allied healthcare professionals who had completed a 2-day training to follow a pre-defined treatment support programme [22]. Monthly for 12 months they: (a) assessed disease activity; (b) reviewed drug treatment; (c) modified drug treatment using a decision tool reflecting “shared treatment plans” formulated with patients during their first visit; and (d) provided supportive psychosocial care.

Intensive Management spanned four strands: (i) providing information about RA with a handbook outlining treatments, side effects and ways to cope with RA; (ii) optimising drug treatment with csDMARDs and biologics using a treatment algorithm; (iii) giving intra-muscular glucocorticoids if arthritis not fully controlled; and (iv) providing “treatment support” focussing on pain and fatigue management; physical activity; medication adherence, sleep and mood. Treatment support used techniques taken from motivational interviewing (MI) [23, 24]. Intensive management consultations were audio recorded by rheumatology practitioners with patients’ consent. A 10% sample of all recorded consultations were assessed against a fidelity checklist developed for TITRATE [12] to monitor the delivery of MI techniques [25].

*Primary Outcome:* DAS28-ESR remission (DAS28-ESR <2.6) at 12 months [26].

*Secondary Outcomes:* alternative measures of remission (DAS28-CRP <2.6, SDAI≤3.3, CDAI≤ 2.8 and ACR/EULAR Boolean remission) [26, 27]) and low disease activity (DAS28-ESR ≤3.2) at 12 months; tender (28/68) and swollen joint counts (28/66); erythrocyte sedimentation rate (ESR); C-reactive protein (CRP); patient global and assessor global assessments on 100 mm visual analogue scales (VAS); pain and fatigue on 100 mm VAS; health assessment questionnaire (HAQ) [28]; EuroQoL 5 Dimensional score (EQ5D-5 L) [29]; plain-film X-rays of hands and feet scored using modified Larsen's scores [30]; NHS and personal social service costs measured by modified Client Service Receipt Inventory (CSRI) questionnaires [31].

### Assessments

An anonymised electronic data capture system collected clinical data. Demographic measures were recorded at baseline. Clinical outcomes were assessed 6- monthly; X-rays were taken and assessed annually.

### Sample size

The most relevant UK treat-to-target trial in active early RA had found 16% of standard care patients achieved end-point DAS remissions [32]. We assumed 16% of standard care patients in TITRATE would similarly achieve endpoint DAS28-ESR remission. We proposed rejecting the null hypothesis (intermediate disease activity RA patients receiving csDMARDs have no more remission after 12-months intensive management) if remission rate increased by ≥15%. Showing this difference with 5% significance level and 90% power indicated the need to randomise 358 patients. Recruitment ended after three years for organisational reasons with 335 patients randomised (94% planned sample size) [12].

### Randomisation

Potentially eligible patients were screened and reasons for non-entry recorded. Consenting patients were individually randomised using block randomisation with randomly varying block sizes. Stratifying by site ensured pre-randomisation allocation concealment. Patients were randomised to intensive management or standard care in a 1:1 ratio. Trial staff were unaware of the allocation sequence.

### Blinding

TITRATE was un-blinded; patient involvement in intensive management made blinding impossible. Independent assessors uninvolved in managing trial patients undertook follow up clinical assessments. Pain, fatigue, disability and quality of life were self-assessed by patients. X-ray reading was performed blinded to treatment.

### Statistical methods

Randomised patients who received treatment were assessed on an intention-to-treat (ITT) basis following CONSORT guidelines [33]. All participants had complete observations at baseline. Missing observations during follow-up (Supplementary Table 1) were multiply imputed regardless of the reason(s) they were missing. Predictive mean matching with five nearest neighbours, assuming unobserved measurements were missing at random was used to impute primary and secondary outcomes. Sensitivity analysis assessed the robustness of the missing at random assumption using pattern-mixture model approach (Supplementary Table 2); it showed qualitatively similar results; consequently, only the primary multiple imputation analyses are reported. A complete case analysis was also undertaken.

Logistic regression analysis was used to evaluate the effect of Intensive Management treatment on the primary outcome (12-month remission) and other binary outcomes. “Univariable” analyses of treatment were adjusted for NHS region (design variable). “Multivariable” analyses further adjusted for gender, ethnicity, age and disease duration. Linear regression evaluated change from baseline to 12-months for the continuous outcomes. Linear mixed models estimated the effect of treatment over follow-up time; working correlation matrices were unstructured as measurements were taken at three time points (i.e. baseline, 6 and 12 months). Interactions between time and treatment group were assessed in these models and were found not to be significant at 5% level and thus the main effect of treatment is reported in the various linear mixed effects analyses after dropping the time by treatment interaction. Serious and other adverse events were evaluated using comparisons of two independent proportions. Analyses were undertaken using Stata 16 [34].

### Economic analyses

NHS and personal social service costs measured from patient resource use questionnaires and NHS hospital records; and adverse events. Total costs and quality-adjusted life years were measured using the EuroQol and combined to assess the health economic effects of intensive management compared with standard care, which was represented by an incremental cost-effectiveness ratio, see (Supplementary Table 3).

### Ethics approval

Ethical approval for this trial was obtained from the London – West London & GTAC

National Research Ethics Service (NRES) Committee (13/LO/1308). All participants provide written informed consent before participating in the trial or extension study.

## Results

### Patients and treatments

**Fig. 1:**
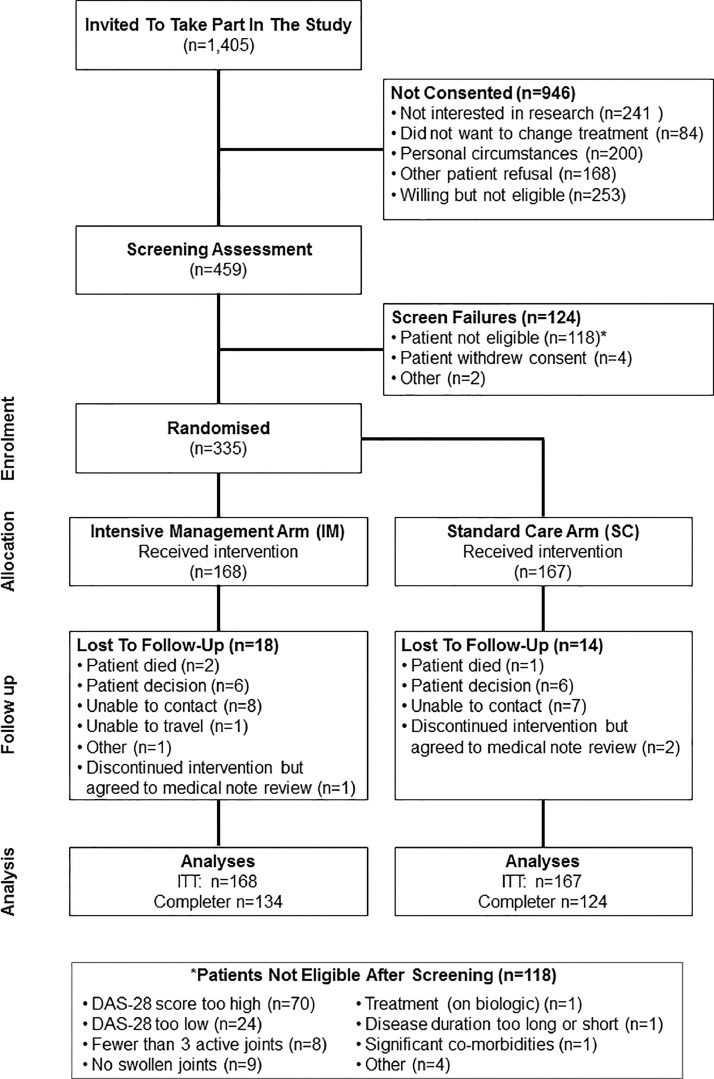
CONSORT diagram for TITRATE Trial.

*Recruitment:* Between August 2014 and July 2017, 1405 patients were invited to participate: 459 patients were screened; 335 patients were randomised and treated (Fig. 1). 303/335 (90%) randomised patients provided 12-month primary outcomes, including 3 patients who withdrew but agreed to medical reviews (Fig. 1).

*Baseline Data and Numbers Analysed:* demographic and disease assessments were similar in both groups (Table 1). The intention to treat analysis included all 335 randomised patients (168 intensive management/167 standard care); the complete case analysis assessed 258 patients (134 intensive management/124 standard care).

**Table 1:** Baseline Characteristics, Assessments And Treatments.

Assessments		Intensive Management	Standard Care
		*n* *=* *168*	*n* *=* *167*
*Demographic*	Age (Years)	56.4 (12.2)	56.8 (12.0)
	Disease Duration (Years)	6.6 (7.0)	5.2 (5.5)
	Female (%)	140 (83%)	130 (78%)

*Clinical Assessments*	DAS28-ESR	4.4 (0.5)	4.3 (0.5)
	DAS28-CRP	4.5 (0.6)	4.5 (0.6)
	CDAI	19.7 (6.5)	20.4 (6.8)
	SDAI	20.6 (6.3)	21.1 (6.6)
	Tender Joint counts (68 joints)	12 (9)	13 (9)
	Swollen joint counts (66 joints)	6 (5)	5 (4)
	Erythrocyte Sedimentation Rate (mm/hr)	18 (14)	15 (13)
	C-Reactive Protein (mg/L)	8 (11)	7 (8)
	Assessor Global Rating (mm)	39 (18)	41 (18)
	Patient Global Assessment (mm)	43 (19)	46 (21)
	Fatigue VAS (mm)	59 (25)	52 (25)
	Pain VAS (mm)	40 (23)	43 (23)
	Health Assessment Questionnaire	1.2 (0.7)	1.2 (0.7)
	EQ5D-5L	0.71 (0.16)	0.70 (0.19)
	Larsen Score	11 (17)	9 (11)

*Drug Treatments*	Oral Methotrexate	59 (35%)	67 (40%)
	Subcut Methotrexate	22 (13%)	19 (11%)
	Sulfasalazine	30 (18%)	19 (11%)
	Leflunomide	12 (7%)	11 (7%)
	Hydroxychloroquine	29 (17%)	37 (22%)
	Azathioprine	1 (1%)	–
	Oral Methotrexate/Hydroxychloroquine	7 (4%)	8 (5%)
	Oral Methotrexate/Sulfasalazine	2 (1%)	1 (1%)
	Subcut Methotrexate/Hydroxychloroquine	3 (2%)	2 (1%)
	Subcut Methotrexate/Sulfasalazine	2 (1%)	–
	Sulfasalazine/Hydroxychloroquine	1 (1%)	3 (2%)

Showing mean (standard deviation) or number (%). Subcut=subcutaneous; ESR= Erythrocyte Sedimentation Rate; CDAI= Clinical Disease Activity Index; SDAI= Simple Disease Activity Index; VAS= Visual Analogue Scale; DAS28-CRP= Disease Activity Score For 28 Joints Based On C-Reactive Protein; DAS28-ESR= Disease Activity Score for 28 Joints based on ESR; EQ5D-5L= EuroQol 5 Dimension (5 levels).

*Baseline Treatments:* all patients took at least one csDMARD; 15/168 (9%) of intensive management patients and 14/168 (8%) of standard care patients took two. Methotrexate, the main csDMARD, was taken by 81 intensive management and 86 standard care patients and as part of csDMARD combinations in another 14 and 11 patients respectively (Table 1).

*Standard Care arm:* 128 patients started another csDMARD, 35 a second and 2 a third. csDMARD doses were increased in 32 patients and decreased in 9. Biologics were started in 24 patients; 2 had a second biological. biological doses were not increased in any patient and were reduced in one during the study period. Depot steroid injections were given to 50 patients: 28 received one injection; 19 received 2–4 injections and 3 had 5 or more injections (Table 2).

**Table 2:** Additional Treatments During Trial Follow Up.

	Intensive Management					Standard Care				
Additional drugs	Oral MTX	Subcut MTX	SSZ	LEF	HCQ	Oral MTX	Subcut MTX	SSZ	LEF	HCQ
	*n* *=* *68*	*n* *=* *27*	*n* *=* *31*	*n* *=* *12*	*n* *=* *29*	*n* *=* *76*	*n* *=* *21*	*n* *=* *22*	*n* *=* *11*	*n* *=* *37*
None	13 (19%)	4 (15%)	3 (10%)	–	2 (7%)	20 (26%)	5 (24%)	7 (32%)	3 (27%)	–
One DMARDs	23 (34%)	12 (44%)	12 (39%)	3 (25%)	5 (17%)	33 (43%)	12 (57%)	9 (41%)	5 (45%)	18 (49%)
Two DMARDs	13 (19%)	6 (22%)	11 (35%)	5 (33%)	9 (31%)	14 (18%)	1 (5%)	5 (23%)	–	11 (30%)
Etanercept	16 (24%)	4 (15%)	4 (13%)	4 (42%)	9 (31%)	4 (5%)	1 (5%)	1 (4%)	2 (18%)	4 (11%)
Benepali	1 (1%)	1 (4%)	1 (3%)	–	1 (4%)	2 (3%)	–	–	–	–
Other TNF Inhibitor	2 (3%)	–	–	–	3 (10%)	3 (4%)	2 (10%)	–	1 (9%)	4 (11%)

Patient who had Azathioprine at baseline also had additional HCQ; SSZ= Sulfasalazine; HCQ= Hydroxychloroquine; MTX= methotrexate; LEF= Leflunomide; DMARD=disease-modifying antirheumatic drugs.

*Intensive Management arm:* 161/168 patients randomised to intensive management attended ≥1 session, with 7 missing all sessions: 3 changed from intensive management to standard care after their first visit; 4 withdrew from the study and were lost to follow up. 139/161(86%) patients attended at least 8 of the planned sessions (mean 11, SD 1.34) and 22/161 (14%) patients attended fewer than 8 visits (mean 4, SD 1.94).

One hundred and forty patients started another csDMARD, 64 a second and 3 a third. csDMARD doses were increased in 69 patients and decreased in 15. Biologics were started in 46 patients: 7 had a second biological and 2 had a third biological. biological doses were increased in 2 patients and reduced in two during the study period. Depot steroid injections were given to 72 patients: 22 received one injection; 33 received 2–4 injections and 17 ≥ 5 injections (Table 2).

A total of 126 sessions were assessed for fidelity to the MI techniques. The data represented that of 42 patients across 19 research sites and 25 trained rheumatology practitioners. Fidelity assessments demonstrated 4 (3%) had poor fidelity to the taught techniques, 52 (41%) low fidelity, 58 (46%) moderate fidelity, and 12 (10%) high fidelity [25]. However, some approaches were undertaken with moderate or high fidelity, such as affirming the patient's strengths and abilities were observed at moderate or high-fidelity levels.

### Primary outcome

Intensive management increased 12-month DAS28-ESR remissions (Table 3) compared to standard care [32% (95% CI: 25%, 40%) vs 18% (12%, 24%)]. The differences were significant in both unadjusted and adjusted logistic regression analyses (*P*<0.01).

**Table 3:** Remission rates with Intensive Management in Intention to Treat Population. The groups were compared using unadjusted and adjusted odds ratios.

Remission Classification	Intensive Management	Standard Care	Unadjusted		Adjusted*	
	Proportion (95% CI)	Proportion (95% CI)	OR (95% CI)	*P*-value	OR (95% CI)	*P*-value
DAS28-ESR	32% (25%, 40%)	18% (12%, 24%)	2.17 (1.28, 3.68)	0.004	2.38 (1.36,4.17)	0.002
DAS28-CRP	21% (15%, 27%)	10% (5%, 15%)	2.44 (1.27, 4.70)	0.008	2.52 (1.28,4.99)	0.008
SDAI	17% (11%, 23%)	10% (6%, 15%)	1.81 (0.94, 3.47)	0.074	1.90 (0.97,3.72)	0.060
CDAI	18% (12%, 24%)	10% (6%, 15%)	1.92 (1.00, 3.68)	0.049	2.10 (1.07,4.09)	0.030
ACR/EULAR Boolean	13% (8%, 18%)	6% (2%, 10%)	2.32 (1.04, 5.18)	0.040	2.44 (1.06,5.64)	0.036

⁎ Adjusted for demographics (age, gender, ethnicity, disease duration), design factors (NHS region) and baseline values; ACR/EULAR Boolean remissions were only adjusted for demographics; standard of care arm was the reference group; OR = odds ratio; CI = confidence intervals.

### Other remissions and low disease activity

There were greater proportions of 12-month remissions in DAS28-CRP, SDAI, CDAI and ACR/EULAR Boolean with intensive management (21%, 17%, 18% and 13%) than standard care (10%, 10%, 10% and 6%) (Table 3).

Low disease activity states at 12 months were achieved by 48% (39%, 56%) on intensive management and in 32% (25%, 40%) of standard care patients. The difference was statistically significant [unadjusted odds ratio 1.94 (1.22, 3.10) *P* = 0.005], [adjusted odds ratio 2.04 (1.25, 3.31) *P* = 0.004].

Mean changes in the DAS28-ESR scores at 12 months from baseline were lower −0.57 (95% CI: −0.88, −0.26; *P*=<0.001) in unadjusted; −0.51 (−0.81, −0.21; *P* = 0.001) in adjusted regression analyses with intensive treatment (Table 4). There were significant differences in DAS28-CRP, SDAI and CDAI 12-months change scores, tender and swollen joint counts, and assessor and patient global 12-months change scores between treatment arms. However mean ESR and C-reactive protein levels were unchanged during the trial without significant difference between groups at 12 months. There was only a small improvement in disability as assessed by HAQ and quality of life assessed by mean EQ5D; the difference between groups were not significant (Table 4).

**Table 4:** Clinical Assessments At 12 Months In Intention To Treat Population. Estimated treatment effects are shown between trial arms.

Assessment	Intensive Management	Standard Care	Linear Regression*				Mixed Effect Models			
	*N* *=* *168*	*N* *=* *167*	Unadjusted	*P*-value	Adjusted	*P*-value	Unadjusted	*P*-value	Adjusted	*P*-value
	Mean (SE)	Mean (SE)	Coefficients		Coefficient		Coefficients		Coefficient	
			(95% CI)		(95% CI)		(95% CI)		(95% CI)	
DAS28-ESR	3.4 (0.1)	3.8 (0.1)	−0.6 (−0.9, −0.3)	<0.001	−0.5 (−0.8, −0.2)	0.001	−0.4 (−0.7, −0.2)	0.001	−0.4 (−0.6, −0.1)	0.003
Tender Joints	7.5 (0.7)	10.8 (0.8)	−2.4 (−4.4, −0.3)	0.023	−2.7 (−4.5, −0.8)	0.004	−1.4 (−3.4, 0.7)	0.187	−1.7 (−3.5, 0.2)	0.076
Swollen joints	3.5 (0.4)	4.9 (0.5)	−1.9 (−3.0, −0.7)	0.002	−1.6 (−2.7, −0.5)	0.004	−1.5 (−2.6, −0.5)	0.005	−1.3 (−2.3, −0.4)	0.006
ESR	17 (1)	15 (1)	−1.5 (−3.9, 1.0)	0.239	−1.1 (−3.4, 1.1)	0.329	−1.1 (−3.2, 1.0)	0.312	−0.7 (−2.7, 1.2)	0.463
CRP	9 (2)	7 (1)	0.9 (−2.6, 4.4)	0.628	1.5 (−1.8, 4.7)	0.372	0.6 (−2.0, 3.1)	0.666	1.3 (−0.9, 3.5)	0.239
Assessor Global	23 (2)	31 (2)	−6 (−12, −0.2)	0.043	−8 (−13, −3)	0.003	−4 (−9, 2)	0.169	−5 (−10, −1)	0.015
Patient Global	29 (2)	41 (2)	−9 (−15, −2)	0.010	−11 (−17, −6)	<0.001	−6 (−12, −1)	0.026	−9 (−14, −4)	<0.001
Fatigue	40 (2)	50 (2)	−18 (−24, −11)	<0.001	−15 (−21, −9)	<0.001	−16 (−21, −10)	<0.001	−13 (−18, −8)	<0.001
Pain	28 (2)	37 (2)	−6.5 (−13., 0.4)	0.064	−8.4 (−15, −2.3)	0.007	−4 (−11, 2)	0.161	−6 (−12, −1)	0.015
HAQ	1.0 (0.1)	1.1 (0.1)	−0.1 (−0.2, 0.0)	0.055	−0.1 (−0.2, 0.0)	0.046	−0.1 (−0.1, 0.0)	0.136	−0.1 (−0.2, 0.0)	0.137
EQ5D-5L	0.76 (0.02)	0.72 (0.02)	0.02 (−0.02, 0.06)	0.248	0.03 (−0.01, 0.07)	0.078	0.02 (−0.01, 0.05)	0.275	0.02 (−0.01, 0.05)	0.121
Larsen score	13 (1)	10 (1)	0.5 (−0.1, 1.0)	0.095	0.4 (−0.2, 0.9)	0.175	–	–	–	–

⁎ Change from baseline analysed and adjustments made for demographics (age, gender, ethnicity, disease duration) design factors (NHS region) and baseline score; standard of care arm was the reference group; SE =standard errors; CI= 95% confidence Intervals.

Mean pain and fatigue scores were significantly lower with intensive management in unadjusted and adjusted linear regression analyses (Table 4). Clinically meaningful improvements in fatigue (10 units or more) were achieved by 58% (95% CI: 51%, 66%) of patients receiving intensive management and 35% (95% CI: 28%, 42%) of patients receiving standard care; logistic regression showed this difference was significant [adjusted odds ratio 2.81 (1.76, 4.48) *P*<0.001]. Larsen X-ray scores increased from mean 11 to 13 with intensive management and 9 to 10 with standard care; with no significant differences between groups (Table 4).

### Longitudinal clinical outcomes

Longitudinal analyses (over the three time points) assessed the overall impact of treatment over time using mixed effects models (Table 4). Unadjusted and adjusted analyses showed significant differences between treatment groups for DAS28-ESR, swollen joint counts for 66 joints, patient global assessments, fatigue and pain. The treatment effects on fatigue between groups were particularly large; −15.7 (95% CI: −21.3, −10.1) in unadjusted and −13.1 (−18.1, −8.1) in adjusted analyses.

### Complete case analyses

The effect of intensive management on remission and clinical outcomes was maintained in the complete case analyses (data not shown). Additional regression analyses showed no evidence that the use of steroids or biologics had an identifiable impact on 12-month remission rates or mean DAS28-ESR, pain or fatigue

An additional exploratory analysis showed that patients who had 11 or 12 intensive management visits achieved the best outcomes in terms of remissions and falls in 12-month DAS28-ESR, pain and fatigue scores (Online Supplementary Figure).

### Adverse events

There were 26 serious adverse events involving 24 patients: 15 with intensive management and 11 with standard care (Table 4); there was no significant difference in the proportion of serious adverse events between treatment groups (Chi-squared=0.64; DF=1; *P* = 0.42). Three patients died: two on intensive management and one on standard care; no death was considered treatment related. Other serious adverse events spanned several systems; there was no indication any were treatment related (Table 5).

**Table 5:** Adverse Events In TITRATE Trial.

Category	Body System	Intensive Management	Standard Care
*Deaths*	Cardiovascular	Ruptured thoracic aneurysm	–
	Neoplasia	Metastatic cancer	–
	Respiratory	–	Pulmonary fibrosis

*Other Individual Serious Adverse Events*	Allergy	–	Angioedema
	Cardiovascular	Heart failure	Microvascular angina
		Myocardial Infarction	Paroxysmal arrhythmia
		Dyspnoea/chest tightness	–
		Hypotension headache	–
	Gastrointestinal	Small bowel obstruction	Diverticular disease
		–	Diverticulitis
		–	Gallstones
	Neoplasia	–	Breast cancer
	Immunological	Tonsillitis with neutropenia	–
	Musculoskeletal	RA flare/shoulder capsulitis	–
	Neurological	Stroke	Sepsis
	Other	Pregnant	Dizziness/syncope
		Collapsed unknown cause	–
		Cerebral spinal fluid leak	–
	Respiratory	Chest infection/Asthma	Exacerbation of Asthma

*All Other Adverse Events*	Number	114 Episodes	151 Episodes
	Allergies	1 (1%)	3 (2%)
	Dermatological	8 (7%)	17 (11%)
	Cardiovascular	5 (4%)	8 (5%)
	Eyes, Ear, Nose & Throat	10 (9%)	15 (10%)
	Gastro-Intestinal	9 (8%)	27 (18%)
	Genitourinary/Renal	3 (3%)	10 (7%)
	Haematological	5 (4%)	3 (2%)
	Hepatic	6 (5%)	2 (1%)
	Immunological	2 (2%)	1 (1%)
	Musculoskeletal	21 (18%)	17 (11%)
	Neoplasia	1 (1%)	3 (2%)
	Neurological	11 (10%)	6 (4%)
	Other	10 (9%)	8 (5%)
	Psychological	0 (0%)	2 (1%)
	Respiratory	22 (19%)	29 (19%)

Overall, 132 patients (60 intensive management; 72 standard care) had 265 adverse events (114 intensive management; 151 standard care) (Table 5). There was no evidence intensive management increased adverse event risks; in fact, a smaller proportion of patients with adverse events and a lower frequency of adverse events were reported in the intensive management arm.

### Cost-Effectiveness

Economic analysis showed the base case incremental cost-effectiveness ratio was £43,972 (€51,474) from medical and personal social service cost perspectives; the probability of meeting the English willing to pay threshold (£30,000/€35,000) was 17%. The incremental cost-effectiveness ratio fell to £29,363 (€24,384) after including patients’ personal costs and lost working time; this corresponded to 50% probability intensive management is cost-effective at English willing to pay thresholds, see (Supplementary Table 4).

## Discussion

The TITRATE trial showed that intensive management using treat-to-target principles in patients with established moderate RA gave substantially more remissions than standard care and more patients achieved a low disease activity state. Intensive management improved joint counts, global assessments of disease activity, fatigue and pain compared with standard care, though neither ESR nor C-reactive protein level were seen to fall with intensive management. There was no evidence intensive management led to more harms. There were numerically more serious adverse events with intensive management than standard care but fewer other adverse events with no significant differences between groups. Although we cannot be certain that in a large study the difference in serious adverse events may be significant, we think this is unlikely because previous systematic reviews of combination DMARD therapies[35] and treat to target trials [8] showed no increases in adverse events in many of these trials.

Economic analysis showed that the incremental cost-effectiveness from a medical and personal social service cost was above current willing to pay thresholds for medical cost in England. That said, our analysis was based on the historic cost of biological treatment when the trial was undertaken; biological acquisition costs have halved since biosimilars were introduced making the current cost-effectiveness of intensive management much more acceptable. The recent move towards telephone and video consultations as a consequence of the Coronavirus Pandemic will also decrease the costs of intensive management. In addition, taking account of patients’ costs and the loss of working time also makes intensive management far more cost-effective.

In strategy trials like TITRATE, patients receive many interventions, with uncertainty about which of them contribute most benefit. Changing csDMARDs, starting biologics and providing psychosocial care may all have contributed; we cannot know which was most important. However, as a recent systematic review of different nurse interventions showed no evidence they specifically reduced disease activity compared to standard care [36], it is likely that patients benefit most when intensive drug treatment is combined with psychosocial care.

There has been debate about combining csDMARDs: some studies and systematic reviews support their use [35, 37]; other studies and guidelines question their value [38, 39]. The use of csDMARDs and biologics in TITRATE cannot resolve this controversy but puts their use into context. Furthermore, forty-five percent of assessed IM consultations demonstrated low/poor fidelity to the taught psychosocial care techniques. Another uncertainty is the frequency of patient assessments. TITRATE aimed for monthly intensive management sessions but less frequent sessions and/or incorporating email/skype consulting methods [40] may have been sufficient – and more cost effective. Our exploratory analysis of the impact of the number of sessions of intensive management suggests infrequent assessments appear suboptimal.

TITRATE had two main strengths. First, as a relatively large trial, involving 39 centres, its findings are robust. Second, the predicted and the actual outcomes were similar, showing it to have delivered the expected clinical improvements [32]. TITRATE had several limitations. Firstly, it did not compare the sustainability of remission between groups [41, 42]: however, assessing standard care patients more often than 6-monthly would mean they were no longer receiving standard care, invalidating them as controls. Secondly, TITRATE only lasted 12 months: ideally strategy trials would last longer; 10-year results have been reported for the BeSt strategy trial [43]. But the organisational and funding complexities of long-term follow up made this impractical in TITRATE. Thirdly, there is uncertainty about which outcome is preferable: ACR-EULAR Boolean remissions appear ideal but are rarely achieved; low DAS28-ESR has less benefit but was achieved by almost half the intensive management patients. Finally, intensive management is not always effective; TITRATE was not designed to show how to manage non-responders. Failure to respond to intensive treatment, particularly biologics, is common and incurs high healthcare costs.

The intensity of RA drug treatment continues to increase, with more combination csDMARDs and biologics used [10, 11]. This increase resulted in 79% standard care TITRATE patients receiving csDMARDs combinations or biologics; only 21% had DMARD monotherapy. The baseline csDMARDs used reflected English practice at the time, with many patients starting sulphasalazine as an initial csDMARD [44], and patients needed to receive two different csDMARDs before starting biological treatments. By contrast the previous TICORA trial [32] showed that 88% standard care patients had DMARD monotherapy. The increasing treatment intensity makes it challenging to compare strategy trials completed years apart. However, unlike the recent negative results from other trials [14] implementing a treat-to-target approach, by combining health care professionals before they delivered intensive management and by providing psychosocial support together with increasing drug treatment, we found intensive management approaches increase remissions in the modern era.

The findings in TITRATE highlight several uncertainties. Firstly, identifying why many patients do not respond to intensive management; analysis of baseline assessments in TITRATE and similar trials may help identify potential non-responders. Secondly, there is limited information about the optimal duration of intensive management strategies in patients who have responded but not achieved sustained remissions. Long-term follow-up of a previous intensive treatment trial suggests benefits decline over time [45], implying persisting intensive management may be needed. Thirdly, simple blood tests like the ESR did not help monitor responses in TITRATE. This finding is in keeping with previous research by Kay and colleagues, who reported that ESR levels often failed to correlate with disease activity measured by joint counts and global assessments in a large observational study [46]; alternative strategies, potentially using multiple measures, may be preferable [47].

We conclude intensive management using treat-to-target approaches benefits patients with moderate established RA as well as patients with active early disease who have been extensively studied in previous trials. TITRATE therefore supports extending treat-to-target approaches to most RA patients who are not yet in remission or low disease activity states. It also highlights the growing importance of non-pharmacological interventions such as psycho-social support. Although our economic analysis showed intensive management exceeded the cost-effective threshold ranges currently used by UK decision makers (≤£30,000/quality-adjusted life-year gained), this estimate reflected historic biological prices at the time of the trial. Adopting current drug acquisition costs would increase the probability intensive management is cost-effective; estimating the impact of remissions after the trial ended would also increase apparent cost-effectiveness.

## Funding

This study was funded by the National Institute for Health Research (NIHR) [Programme Grants for Applied Research (Grant Reference Number RP-PG-0610–10,066)]. The views expressed are those of the authors and not necessarily those of the NIHR or the Department of Health and Social Care.

Dr Brian Tom is supported by the UK Medical Research Council (Unit Programme number MC_UP_1302/3 & MRC_MC_UU_00002/2).

## Data monitoring committee

Dr Richard Watts (Chair)

Dr Kimme Hyrich

Dr Mark Lunt

## Trial steering committee and programme grant steering committee

Professor David G Scott (Chair)

Professor Anisur Rahman

Professor James Ritter

Dr Louise Pollard

Luke Brewer

Jo Cumming

Chris Ward

Federico Muscoguiri

Kirandeep Chana

Quang Tan Ho

Judith Rosheuvel

Celia Manson

Carrie Gibbens

Dr Aneela Mian

Dr Lindsay Bearne

Joy Ellery

Dr Nicola Gullick

Dr Karen Walker-Bone

Professor Anthony Woolf

Deborah Johnson

Professor Ewan Ferlie

Carol Simpson

## Data sharing statement

The data generated during the trial are not publicly available because data sharing would require institutional approval. Anonymised summary data will be available from the corresponding author for inclusion in meta-analyses and other relevant similar academic endeavours

## Author contributors

The TITRATE programme investigators contributing authors have (1) substantially contributed to the design of the trial and were co-applicant of the grant; and (2) have lead the work packages within the programme grant; the analysis of the data; interpretation of data; and have been involved in the (3) drafting the paper or revising it critically; and have given (4) the final approval of the version to be published.

## Declaration of Competing Interest

A list of investigators with competing interests and the details they have declared is as follows: David L Scott has received funding from Novartis Pharmaceuticals UK Limited for advice on using biosimilars. Dr James Galloway has received personal fees and non-financial support from Abbvie, personal fees from BMS, grants and personal fees from Celgene, personal fees from Janssen, grants, personal fees and non-financial support from Pfizer, personal fees from UCB. Dr Elena Nikiphorou has received speaker honoraria/participated in advisory boards for Pfizer, AbbVie, Sanofi, Gilead, Celltrion and Lilly. Professor Jackie Sturt has received personal fees from Eli Lilly and grants and personal fees from Spirit Healthcare. The following authors declare that they have no competing interests: Dr Naomi Martin, Fowzia Ibrahim, Dr Brian Tom, Professor Allan Wailoo, Dr Harry Hill, Dr Louise Prothero, Dr Rhiannon R Baggott, Dr Sofia Georgopoulou, Ailsa Bosworth, Isabel Neatrour, Professor Frances MK Williams, Dr Ruth Williams and Dr Heidi Lempp.

## Appendix Supplementary materials

Image, application 1

Image, image 2

## References

[1] Scott D.L., Wolfe F., Huizinga T.W (2010). Rheumatoid arthritis. Lancet.

[2] Smolen J.S., Aletaha D., McInnes I.B (2016). Rheumatoid arthritis. Lancet.

[3] van der Woude D., van der Helm-van Mil A.H.M (2018). Update on the epidemiology, risk factors, and disease outcomes of rheumatoid arthritis. Best Practice Res. Clinical Rheum..

[4] N.I.C.E.Rheumatoid arthritis: The management of rheumatoid arthritis in adults: national Institute for Health and Clinical Excellence; 2009.

[5] Smolen J.S., Breedveld F.C., Burmester G.R., Bykerk V., Dougados M., Emery P. (2016). Treating rheumatoid arthritis to target: 2014 update of the recommendations of an international task force. Ann. Rheum. Dis..

[6] Schoels M., Knevel R., Aletaha D., Bijlsma J.W.J., Breedveld F.C., Boumpas D.T. (2010). Evidence for treating rheumatoid arthritis to target: results of a systematic literature search. Ann. Rheum. Dis..

[7] Stoffer M.A., Schoels M.M., Smolen J.S., Aletaha D., Breedveld F.C., Burmester G. (2016). Evidence for treating rheumatoid arthritis to target: results of a systematic literature search update. Ann. Rheum. Dis..

[8] Wailoo A., Hock E.S., Stevenson M., Martyn-St James M., Rawdin A., Simpson E. (2017). The clinical effectiveness and cost-effectiveness of treat-to-target strategies in rheumatoid arthritis: a systematic review and cost-effectiveness analysis. Health Technol. Asses..

[9] Hughes C.D., Scott D.L., Ibrahim F (2018). Intensive therapy and remissions in rheumatoid arthritis: a systematic review. BMC Musculoskelet Disord..

[10] Mian A.N., Ibrahim F., Scott I.C., Bahadur S., Filkova M., Pollard L. (2016). Changing clinical patterns in rheumatoid arthritis management over two decades: sequential observational studies. BMC Musculoskelet Disord..

[11] Gullick N.J., Ibrahim F., Scott I.C., Vincent A., Cope A.P., Garrood T. (2019). Real world long-term impact of intensive treatment on disease activity, disability and health-related quality of life in rheumatoid arthritis. BMC Rheumatol..

[12] Martin N.H., Ibrahim F., Tom B., Galloway J., Wailoo A., Tosh J. (2017). Does intensive management improve remission rates in patients with intermediate rheumatoid arthritis? (the TITRATE trial): study protocol for a randomised controlled trial. Trials.

[13] Symmons D., Tricker K., Roberts C., Davies L., Dawes P., Scott D.L (2005). The British Rheumatoid Outcome Study Group (BROSG) randomised controlled trial to compare the effectiveness and cost-effectiveness of aggressive versus symptomatic therapy in established rheumatoid arthritis. Health Technol. Assess..

[14] Harrold L.R., Reed G.W., John A., Barr C.J., Soe K., Magner R. (2018). Cluster-Randomized Trial of a Behavioral Intervention to Incorporate a Treat-to-Target Approach to Care of US Patients With Rheumatoid Arthritis. Arthritis Care Res (Hoboken).

[15] Desai S., Leatherwood C., Forman M., Ko E., Stevens E., Iversen M. (2019). Treat‐to‐target in Rheumatoid Arthritis: a Quality Improvement Trial. Arthritis Care. Res. (Hoboken).

[16] Solomon D.H., Lee S.B., Zak A., Corrigan C., Agosti J., Bitton A. (2016). Implementation of treat-to-target in rheumatoid arthritis through a Learning Collaborative: rationale and design of the TRACTION trial. Semin Arthritis Rheum.

[17] Solomon D.H., Losina E., Lu B., Zak A., Corrigan C., Lee S.B. (2017). Implementation of treat‐to‐target in rheumatoid arthritis through a learning collaborative: results of a randomized controlled trial. Arthritis Rheumatol..

[18] van Vollenhoven R. (2019). Treat-to-target in rheumatoid arthritis—Are we there yet?. Nature Rev. Rheumatol..

[19] Aletaha D., Neogi T., Silman A.J., Funovits J., Felson D.T., Bingham C.O. (2010). 2010 rheumatoid arthritis classification criteria: an American College of Rheumatology/European League Against Rheumatism collaborative initiative. Ann. Rheumatic Diseases..

[20] Allen A., Carville S., McKenna F (2018). Diagnosis and management of rheumatoid arthritis in adults: summary of updated NICE guidance. BMJ (Clinical Research ed).

[21] NICE. Rheumatoid arthritis in adults: management. National Institute for Health and Clinical Excellence.

[22] Georgopoulou S., Prothero L., Lempp H., Galloway J., Sturt J (2016). Motivational interviewing: relevance in the treatment of rheumatoid arthritis?. Rheumatol. (Oxford).

[23] Miller W.R. (1983). Motivational interviewing with problem drinkers. Behav. Cogn. Psychother..

[24] Rollnick S., Miller W.R. (1995). What is motivational interviewing?. Behav. Cogn. Psychother.

[25] Prothero L., Georgopoulou S., Lempp H., Sturt J. (2019). Fidelity assessment of motivational interviewing-based treatment support delivered by nurses. European Health Psychology Society Conference.

[26] Aletaha D., Ward M.M., Machold K.P., Nell V.P., Stamm T., Smolen J.S (2005). Remission and active disease in rheumatoid arthritis: defining criteria for disease activity states. Arthritis Rheum..

[27] Felson D.T., Smolen J.S., Wells G., Zhang B., van Tuyl L.H., Funovits J. (2011). American College of Rheumatology/European League against Rheumatism provisional definition of remission in rheumatoid arthritis for clinical trials. Ann. Rheum. Dis..

[28] Bruce B., Fries J.F. (2003). The Stanford Health Assessment Questionnaire: a review of its history, issues, progress, and documentation. J. Rheumatol..

[29] Group T.E. (1990). EuroQol-a new facility for the measurement of health-related quality of life. Health Policy (New York).

[30] Scott D., Houssien D., Laasonen L (1995). Proposed modification to Larsen's scoring methods for hand and wrist radiographs. Rheumatology.

[31] Chisholm D., Knapp M.R.J., Knudsen H.C., Amaddeo F., Gaite L., Van Wijngaarden B. (2000). Client socio-demographic and service receipt inventory–European version: development of an instrument for international research: EPSILON Study 5. British J. Psychiatry.

[32] Grigor C., Capell H., Stirling A., McMahon A.D., Lock P., Vallance R. (2004). Effect of a treatment strategy of tight control for rheumatoid arthritis (the TICORA study): a single-blind randomised controlled trial. Lancet.

[33] http://www.consort-statement.org/.

[34] Cooperation S. (2017). Stata 15 release.

[35] Hazlewood G.S., Barnabe C., Tomlinson G., Marshall D., Devoe D.J.A., Bombardier C (2016). Methotrexate monotherapy and methotrexate combination therapy with traditional and biologic disease modifying anti‐rheumatic drugs for rheumatoid arthritis: a network meta‐analysis. Cochrane Database Syst. Rev..

[36] Lempp H., Baggott R., Scott D.L., Parker L., Bosworth A., Georgopoulou S. (2020). The value, impact and role of nurses in rheumatology outpatient care: critical review of the literature. Musculoskeletal Care.

[37] Scott D.L., Ibrahim F., Farewell V., O'Keeffe A.G., Walker D., Kelly C. (2015). Tumour necrosis factor inhibitors versus combination intensive therapy with conventional disease modifying anti-rheumatic drugs in established rheumatoid arthritis: TACIT non-inferiority randomised controlled trial. BMJ (Clinical Research ed).

[38] Verschueren P., De Cock D., Corluy L., Joos R., Langenaken C., Taelman V. (2015). Methotrexate in combination with other DMARDs is not superior to methotrexate alone for remission induction with moderate-to-high-dose glucocorticoid bridging in early rheumatoid arthritis after 16 weeks of treatment: the CareRA trial. Ann. Rheum. Dis..

[39] Smolen J.S., Landewe R., Bijlsma J., Burmester G., Chatzidionysiou K., Dougados M. (2017). EULAR recommendations for the management of rheumatoid arthritis with synthetic and biological disease-modifying antirheumatic drugs: 2016 update. Ann. Rheum. Dis..

[40] Griffiths F., Bryce C., Cave J., Dritsaki M., Fraser J., Hamilton K. (2017). Timely digital patient-clinician communication in specialist clinical services for young people: a mixed-methods study (the LYNC study). J. Med. Internet Res..

[41] Ma M.H.Y., Ibrahim F., Kingsley G.H., Cope A., Scott D.L (2018). Variable impacts of different remission states on health-related quality of life in rheumatoid arthritis. Clin. Exp. Rheumatol..

[42] Einarsson J.T., Willim M., Ernestam S., Saxne T., Geborek P., Kapetanovic M.C (2019). Prevalence of sustained remission in rheumatoid arthritis: impact of criteria sets and disease duration, a Nationwide Study in Sweden. Rheumatol. (Oxford).

[43] Markusse I.M., Akdemir G., Dirven L., Goekoop-Ruiterman Y.P., van Groenendael J.H., Han K.H. (2016). Long-Term Outcomes of Patients With Recent-Onset Rheumatoid Arthritis After 10 Years of Tight Controlled Treatment: a Randomized Trial. Ann. Intern. Med..

[44] Kiely P., Williams R., Walsh D., Young A (2009). Contemporary patterns of care and disease activity outcome in early rheumatoid arthritis: the ERAN cohort. Rheumatology.

[45] Burgers L., van der Pol J., Huizinga T., Allaart C., van der Helm-van Mil A (2019). Does treatment strategy influence the ability to achieve and sustain DMARD-free remission in patients with RA? Results of an observational study comparing an intensified DAS-steered treatment strategy with treat to target in routine care. Arthritis Res. Ther..

[46] Kay J., Morgacheva O., Messing S.P., Kremer J.M., Greenberg J.D., Reed G.W. (2014). Clinical disease activity and acute phase reactant levels are discordant among patients with active rheumatoid arthritis: acute phase reactant levels contribute separately to predicting outcome at one year. Arthritis Res. Ther..

[47] Johnson T.M., Register K.A., Schmidt C.M., O'Dell J.R., Mikuls T.R., Michaud K. (2019). Correlation of the Multi‐Biomarker Disease Activity Score With Rheumatoid Arthritis Disease Activity Measures: a Systematic Review and Meta‐Analysis. Arthritis Care Res (Hoboken).

