# Supplementary material for: The clinical effectiveness of intensive management in moderate established rheumatoid arthritis: The titrate trial
Source: Semin Arthritis Rheum. 2020 Oct;50(5):1182–90. doi: 10.1016/j.semarthrit.2020.07.014 (PMC7390769; doi:10.1016/j.semarthrit.2020.07.014)
Supplement: Supplementary file 1 [file mmc1.docx]

**Supplementary Files**

335 patients were randomised and treated. Of the randomised patients, 303/335 (90%) patients provided a primary outcome measure at 12 months, including 3 patients who withdrew but agreed medical review only; 32/335 (10%) patients were lost to follow up.

**Supplementary Table 1. Missing observations in the components of composite outcome (DAS28-ESR) and secondary outcome measures during the trial follow up**

|  | **Intensive Management**  **N=168** | | | **Standard Care**  **N=167** | | | **Total**  **N=335** | | |
| --- | --- | --- | --- | --- | --- | --- | --- | --- | --- |
|  | *Missing 6 months only* | *Missing at 12 months only* | *Missing 6 & 12 months* | *Missing 6 months only* | *Missing at 12 months only* | *Missing 6 & 12 months* | *Missing 6 months only* | *Missing at 12 months only* | *Missing 6 & 12 months* |
| **Primary outcome** |  |  |  |  |  |  |  |  |  |
| Tender and swollen joint counts^¥^ | 0 (0%) | 5 (3%) | 10 (6%) | 2 (1%) | 4 (2%) | 7 (4%) | 2 (1%) | 9 (2.7%) | 17 (5.1%) |
| ESR | 1 (1%) | 7 (4%) | 10 (6%) | 3 (2%) | 7 (4%) | 8 (5%) | 4 (1%) | 14 (4.2%) | 18 (5.4%) |
| Patient Global | 0 (0%) | 6 (4%) | 10 (6%) | 1 (1%) | 3 (2%) | 7 (4%) | 1 (1%) | 9 (2.7%) | 17 (5.1%) |
| DAS28-ESR | 1 (1%) | 8 (5%) | 10 (6%) | 4 (2%) | 7 (4%) | 8 (5%) | 5 (1.5%) | 15 (4.5%) | 18 (5.4%) |
| **Secondary outcomes** |  |  |  |  |  |  |  |  |  |
| Extended Joint counts | 5 (3%) | 5 (3%) | 10 (6%) | 3 (2%) | 6 (4%) | 8 (5%) | 8 (2.4%) | 11 (3.3%) | 18 (5.4%) |
| CRP | 0 (0%) | 6 (4%) | 10 (6%) | 5 (3%) | 4 (2%) | 7 (4%) | 5 (1.5%) | 10 (3.0%) | 17 (5.1%) |
| DAS28-CRP | 0 (0%) | 7 (4%) | 10 (6%) | 6 (4%) | 5 (3%) | 7 (4%) | 6 (1.8%) | 12 (3.6%) | 17 (5.1%) |
| AG | 0 (0%) | 5 (3%) | 11 (7%) | 2 (1%) | 3 (2%) | 9 (5%) | 2 (1%) | 8 (2.4%) | 20 (6.0%) |
| HAQ | 5 (3%) | 12 (7%) | 13 (8%) | 12 (7%) | 9 (5%) | 10 (6%) | 17 (5.1%) | 19 (5.7%) | 23 (6.9%) |
| MARS | 1 (1%) | 6 (4%) | 11 (7%) | 2 (1%) | 4 (2%) | 9 (5%) | 3 (1%) | 10 (3%) | 20 (6.0%) |
| EQ5D-5L | 1 (1%) | 5 (3%) | 11 (7%) | 3 (2%) | 4 (2%) | 9 (5%) | 4 (1.2%) | 9 (2.7%) | 20 (6.0%) |
| Pain VAS | 1 (1%) | 6 (4%) | 11 (7%) | 2 (1%) | 3 (2%) | 9 (5%) | 3 (1%) | 9 (2.7%) | 20 (6.0%) |
| Fatigue VAS | 1 (1%) | 6 (4%) | 11 (7%) | 2 (1%) | 3 (2%) | 9 (5%) | 3 (1%) | 9 (2.7%) | 20 (6.0%) |

ESR= Erythrocyte Sedimentation Rate; DAS28-ESR= Disease Activity Score for 28 Joints based on ESR;VAS= Visual Analogue Scale; CRP=C-Reactive Protein; DAS28-CRP= Disease Activity Score for 28 Joints based on CRP; EQ5D-5L= EuroQol 5 Dimension (5 levels); PG=Patient Global; AG=Assessor Global; MARS= Medication Adherence Rating Scale; HAQ= Health assessment questionnaires; ^¥^Missing tender and swollen joint counts are missing for the same patients

**Supplementary Table 2. Details of Imputation For Missing Data**

| **Imputation Methods** |
| --- |
| Missing primary and secondary outcome measures were imputed using multiple imputation with chained equations and predictive mean matching using five nearest neighbours (1). We assumed the data were missing under the missing at random assumption.  In subjects who had missing outcomes at 6 months, under the monotone assumption, baseline outcomes and explanatory covariates were used to impute the missing values at 6 months.  For patients who had missing outcomes at 12 months, baseline and 6 months outcomes with explanatory covariates were used to impute the missing values at 12 months. If outcome variables were missing at 6 and 12 months, then the outcome variables at 6 months was imputed first followed by the outcomes at 12 months.  The primary endpoint was the composite score DAS28-ESR, the overall composite score was imputed instead of imputing the components of the composite outcome and recalculating the composite score.  The imputation was 20 cycles. At the end of the cycle, one imputed dataset was created. The process was repeated to create 20 imputed datasets. The results from the analyses of the 20 datasets were combined using Rubin’s rules (2, 3) Therefore, the estimates and standard errors presented here are the combined ones. |
| We conducted sensitivity analysis to assess the robustness of the missing at random  assumption using pattern-mixture model approach. We specify Delta (δ), which is the difference between observed and unobserved data, in this case the coefficient corresponds to the log odds ratio for patients with an observed outcome and those patients with outcome missing. We assumed δ to be three points (-3/+3) worse on composite outcome and its components compared to MAR assumption in both trial arms. Stata’s *rctmiss* command written by Ian White was used to analyse the sensitivity analysis (4) |

**Supplementary Table 3. Details of Health Economics Analysis**

**Costs**

NHS resources were measured for each participant between baseline and final follow-up. This included medication costs, visits to health services and any social care and community support. Medication usage, the number of hospital visits and intensive management appointments were taken from trial records. NHS and personal social service resources were self-reported by participants at 6 months and 12 months with the widely used and validated Client Service Receipt Inventory (CSRI) questionnaire (5). It included questions related to time-off-work used in sensitivity analysis. Unit costs for all resources were obtained for the financial year 2018–19 from national sources. Medication use was taken from a form that collected current RA medication information over the trial. This included the duration of the medication (start date and end date of the medication), the dosage and frequency of the medication. NHS unit costs for the medications were based on the drug tariff price reported in the British National Formulary (BNF) (2019) (6). If the drug tariff price was not reported in the BNF we used the average NHS indicative price from all manufactures of the medication. NHS and social services costs were from Unit Costs of Health and Social Care (PSSRU, 2019) (7).

**Economic Outcomes**

For each participant we estimated total costs over the trial period and the QALYs gained. Mean differences between the two trial-arms are presented with sample 95% confidence intervals and bootstrapped bias corrected 95% confidence intervals. The cost and health information were combined in an estimate of cost-effectiveness, the incremental cost-effectiveness ratio (ICER) of intensive vs standard care. Non-parametric bootstrapping was used to generate CIs for the estimated mean incremental costs and effects, and to summarise the uncertainty surrounding the ICERs. Uncertainty was visualised as a two-dimensional cost-effectiveness plane, and as a cost-effectiveness acceptability curve (CEAC) which reports the probability that the intervention is cost-effective for any given level of willingness to pay.

**Sensitivity analysis**

To further test the robustness of results derived from the base-case analysis, several deterministic sensitivity analyses were conducted. This included estimating an ICER with alternative approaches to establishing health utilities (8) and a societal cost perspective that includes NHS and social services costs and productivity losses(9). Such analyses examine the effect of estimated or uncertain parameters on the decision.

**Subgroup analysis**

To explore the sensitivity of the EQ-5D instrument to detect changes in remission we summarise the mean QALY gain at 12 months for participants that achieved remission compared to those that did not. Remission is defined as a DAS28-ESR score of less than 2.6 at 12 months.

**Supplementary Table 4. Economic Outcomes from the Trial Evaluation**

| **Cost Perspective** | **Intensive Management**  **(Bootstrapped Bias Corrected 95% CI)** | **Standard Care**  **(Bootstrapped Bias Corrected 95% CI)** | **Mean Difference** |
| --- | --- | --- | --- |
|  | ***n=168*** | ***n=167*** |  |
| All NHS and personal social service costs | £3784  (£3371, £4246) | £2258  (£1974, £2585) | £1526 |
| Societal cost perspective* | £4697  (£4076, £5378) | £3678  (£2926, £5025) | £1019 |
| QALYs with regression adjustment for baseline EQ5D-3L | 0.64  (0.62, 0.66) | 0.61  (0.58, 0.63) | 0.035  P=0.02 |
| ICER (NHS And Social Services Costs) | | | £43,972 |
| *Sensitivity analysis* | | |  |
| ICER (Societal cost perspective*) | | | £29,363 |
| QALYs estimated using Hernandez et al (2017) mapping function derived from the EuroQol Group coordinated dataset | | |  |
| ICER (NHS and social services costs) | | | £47,293 |
| ICER (Societal cost perspective) | | | £31,580 |
| QALYs estimated using Hernandez et al (2017) mapping function derived from the FORWARD National Databank for Rheumatic Diseases) | | |  |
| ICER (NHS and social services costs) | | | £52,188 |
| ICER (Societal cost perspective) | | | £34,849 |
| QALYs based on EQ-5D-5L index scores | | |  |
| ICER (NHS and social services costs) | | | £57,849 |
| ICER (Societal cost perspective) | | | £38,629 |

*Societal costs include NHS and social services costs and productivity losses

**References**

1. Di Zio M, Guarnera U. Semiparametric predictive mean matching. AStA Advances in Statistical Analysis. 2009;93(2):175-86.

2. Little RJ, Rubin DB. Statistical analysis with missing data: Wiley; 2002.

3. Scheffer J. Dealing with missing data. 2002.

4. White I. RCTMISS: Stata module to analyse a randomised controlled trial (RCT) allowing for informatively missing outcome data. 2018.

5. Chisholm D, Knapp MRJ, Knudsen HC, Amaddeo F, Gaite L, Van Wijngaarden B, et al. Client socio-demographic and service receipt inventory–European version: development of an instrument for international research: EPSILON Study 5. The British Journal of Psychiatry. 2000;177(S39):s28-s33.

6. British National Formulary 2018 [%&].

7. Kent Uo. Personal Social Services Research Unit. 2019.

8. Stevenson MD, Wailoo AJ, Tosh JC, Hernandez-Alava M, Gibson LA, Stevens JW, et al. The Cost-effectiveness of Sequences of Biological Disease-modifying Antirheumatic Drug Treatment in England for Patients with Rheumatoid Arthritis Who Can Tolerate Methotrexate. The Journal of rheumatology. 2017;44(7):973-80.

9. Jönsson B. Ten arguments for a societal perspective in the economic evaluation of medical innovations. Springer; 2009.
