# Supplementary material for: The clinical effectiveness of intensive management in moderate established rheumatoid arthritis: The titrate trial
Source: Semin Arthritis Rheum. 2020 Oct;50(5):1182–90. doi: 10.1016/j.semarthrit.2020.07.014 (PMC7390769; doi:10.1016/j.semarthrit.2020.07.014)
Supplement: Supplementary file 2 [file mmc2.pptx]

## Slide 1
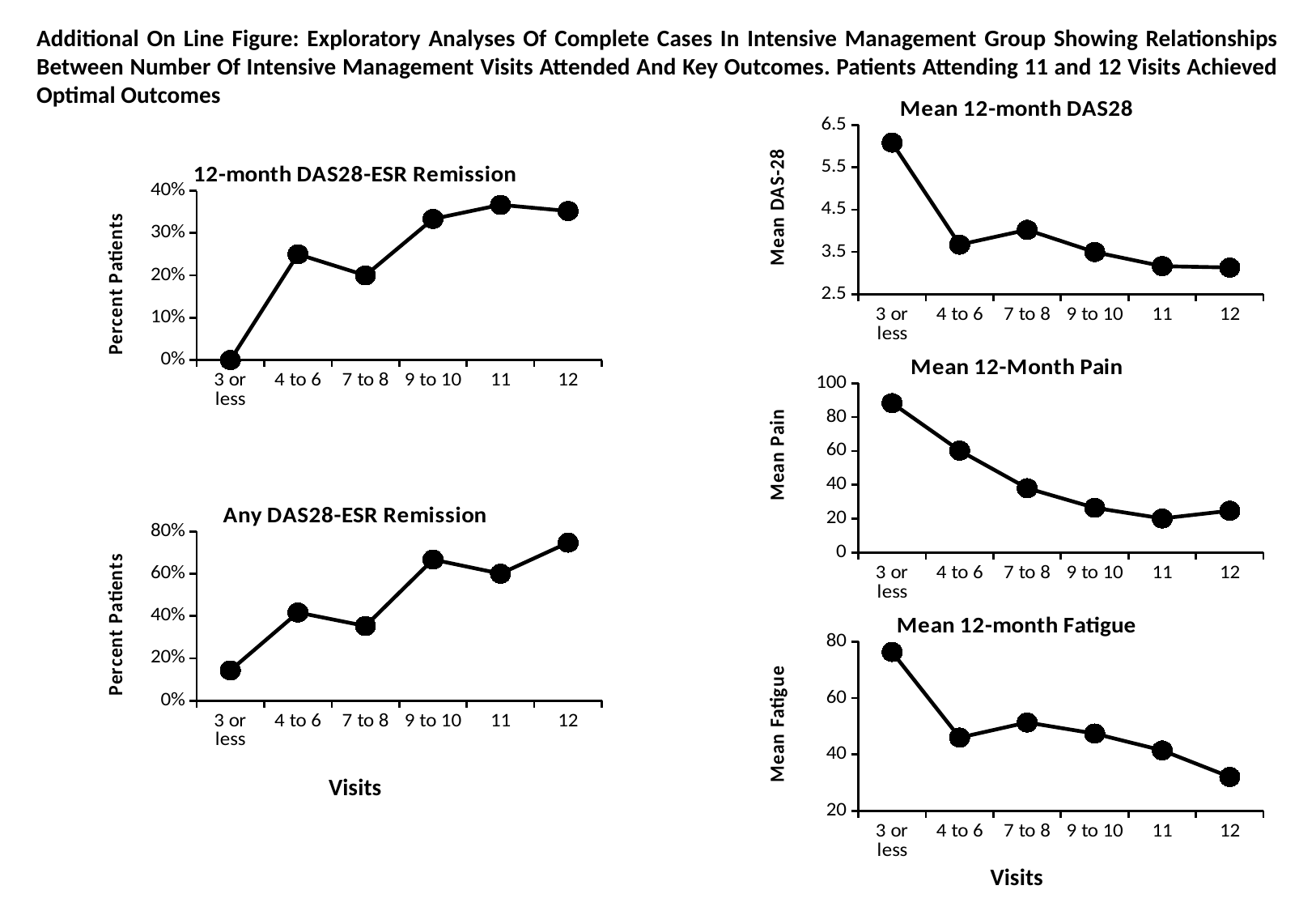

Additional On Line Figure: Exploratory Analyses Of Complete Cases In Intensive Management Group Showing Relationships Between Number Of Intensive Management Visits Attended And Key Outcomes. Patients Attending 11 and 12 Visits Achieved Optimal Outcomes
### Chart:
| Category | Mean 12-month DAS28 |
|---|---|
| 3 or less | 6.083333333333333 |
| 4 to 6 | 3.6725000000000003 |
| 7 to 8 | 4.024 |
| 9 to 10 | 3.4983333333333335 |
| 11 | 3.1653333333333324 |
| 12 | 3.1323761019432608 |
### Chart:
| Category | 12-month DAS28-ESR Remission |
|---|---|
| 3 or less | 0.0 |
| 4 to 6 | 0.25 |
| 7 to 8 | 0.2 |
| 9 to 10 | 0.3333333333333333 |
| 11 | 0.36666666666666664 |
| 12 | 0.352112676056338 |
### Chart:
| Category | Mean 12-Month Pain |
|---|---|
| 3 or less | 88.33333333333333 |
| 4 to 6 | 60.25 |
| 7 to 8 | 38.05882352941177 |
| 9 to 10 | 26.458333333333336 |
| 11 | 20.166666666666668 |
| 12 | 24.742857142857133 |
### Chart:
| Category | Any DAS28-ESR Remission |
|---|---|
| 3 or less | 0.14285714285714285 |
| 4 to 6 | 0.4166666666666667 |
| 7 to 8 | 0.35294117647058826 |
| 9 to 10 | 0.6666666666666666 |
| 11 | 0.6 |
| 12 | 0.7464788732394366 |
### Chart:
| Category | Mean 12-month Fatigue |
|---|---|
| 3 or less | 76.33333333333333 |
| 4 to 6 | 46.0 |
| 7 to 8 | 51.352941176470594 |
| 9 to 10 | 47.41666666666667 |
| 11 | 41.43333333333334 |
| 12 | 32.057142857142864 |Visits
Visits
